# Comparative Evaluation of Surgical Difficulty of Impacted Maxillary and Mandibular Third Molars: An Observational Study

**DOI:** 10.7759/cureus.71356

**Published:** 2024-10-13

**Authors:** Nishtha Glodha, Vijayta Yadav, Gaurav Verma, Surbhi Agarwal, Mohd Zeeshan, Soumitra Agarwal, Seema Gupta

**Affiliations:** 1 Orthodontics, Kothiwal Dental College and Research Centre, Moradabad, IND; 2 Oral Surgery, Kothiwal Dental College and Research Centre, Moradabad, IND; 3 Pediatric Dentistry, Kothiwal Dental College and Research Centre, Moradabad, IND; 4 Oral Medicine and Radiology, Kothiwal Dental College and Research Centre, Moradabad, IND

**Keywords:** difficulty, impacted, index, surgical, third molars

## Abstract

Introduction

Extraction of the third molar is the surgical intervention most frequently performed by dental professionals. Notwithstanding a meticulously designed surgical strategy, complications may arise during extraction of the lower third molars. The primary objective of this study was to evaluate and compare the surgical difficulties encountered when managing impacted maxillary and mandibular third molars. The secondary objective was identifying the predictors of surgical difficulty in extracting the third molars from both arches.

Materials and methods

This study included 30 systemically healthy patients requiring upper and lower third molar extractions on the same side. The Pederson index was used to assess surgical difficulty in such patients on pre-treatment orthopantomograms. The time interval between upper and lower extraction was one week for all patients, and all extractions were performed by an experienced oral and maxillofacial surgeon. An independent t-test was performed to determine the significance of the differences between group variables. Predictors of surgical difficulty, such as sex, type of impaction, duration of surgical procedure, type of surgical procedure, fused roots, and root curvature, were assessed using multivariate analysis.

Results

The mean patient age was 29.93 ± 2.81 years. The mean mouth opening was 41.25 ± 3.5 mm. Chi-square test results revealed no significant differences between the maxillary and mandibular groups across all variables (p > 0.05). The independent t-test results revealed significant differences between the maxillary and mandibular groups in procedure duration (p < 0.05). The duration of the procedure was significantly longer in the mandibular group (44.13 ± 15.20 minutes) than in the maxillary group (33.33 ± 13.96 minutes). Root curvature, duration of the surgical procedure, type of surgical technique employed, and patient sex were all identified as significant predictors.

Conclusion

The surgical difficulty for impacted third molars was similar in both arches. The duration of the surgical procedure was longer in the mandibular arch.

## Introduction

Impacted third molar surgery (ITMS) is a frequently performed procedure in oral surgery. Third molars exhibit a heightened prevalence of developmental anomalies, are ill-suited for soft tissue surroundings, and present challenges in terms of oral hygiene maintenance [1]. ITMS is often indicated in cases of infection, associated cysts or tumors, orthodontic purposes, pericoronitis, and associated resorption of adjacent teeth [1]. Extraction of such teeth has been associated with postoperative complications, such as dry socket, pain, swelling, inferior alveolar nerve damage or injury, and trismus [2].

Similarly, maxillary third molar surgery (MTMS) has been associated with sinus floor perforation, fracture of the maxillary tuberosity, displacement of the root into the maxillary sinus, and the infratemporal or buccal space [3]. Many studies have been conducted to assess the treatment difficulty of mandibular molars [4,5], and the most commonly used index to assess treatment difficulty is the Pederson index [4-8]. Moreover, few studies have been conducted to assess the treatment difficulty for MTMS [9,10], and it has been observed that MTMS difficulty is low [10]. Nevertheless, conducting a thorough preoperative assessment of the treatment complexity for ITMS is crucial, as it aids in predicting challenges, devising surgical strategies, and optimizing scheduling. To the best of our knowledge, no study has been conducted to analyze and compare the level of surgical complexity involved in the treatment of impacted maxillary and mandibular molars. Consequently, the primary objective of this study was to evaluate and compare the surgical difficulties encountered when managing impacted maxillary and mandibular third molars. The secondary objective was to identify the predictors of surgical difficulty in extracting the third molars from both arches. The null hypothesis of this study was that there would be no disparity in the difficulty index between the impacted maxillary and mandibular third molars.

## Materials and methods

Study design and setting

This cross-sectional observational study was conducted in the Department of Oral and Maxillofacial Surgery, Kothiwal Dental College and Research Centre, Moradabad, India, from January 2022 to December 2023. Institutional Ethics and Review Board (IERB) approval was secured (KDCRC/IERB/12/2021/16), and the study adhered to the Strengthening the Reporting of Observational Studies in Epidemiology (STROBE) guidelines for observational studies as well as the principles outlined in the Declaration of Helsinki. Written informed consent was obtained from all patients.

Sample size calculation

The sample size for this study was determined using an 80% power and an alpha error of 5%. Based on a medium effect size, with an expected mean difference in the impacted molar difficulty index of 0.65, an a priori power analysis using G power software version 3.6.9 indicated that 30 patients would be required.

Patients’ participation

The study included 30 patients requiring absolute indication for extraction of both the upper and lower third molars under local anesthesia, falling within categories I and II of the American Society of Anesthesiology, having upper and lower second molars present, normal mouth opening of 35-55 mm, systemically healthy, and providing informed consent. All participants exhibited functional third molars, which were directed by the Orthodontics Department for surgical removal in individuals undergoing preparation for orthodontic intervention. The study excluded patients with systemic conditions that made surgical removal of third molars inadvisable, individuals with allergies to local anesthesia, pregnant or breastfeeding women, individuals who had undergone irradiation, those at high risk for neural injury, patients with a history of complications during prior third molar extractions, and patients with third molars associated with odontogenic cysts and tumors.

Methodology

**Figure 1:**
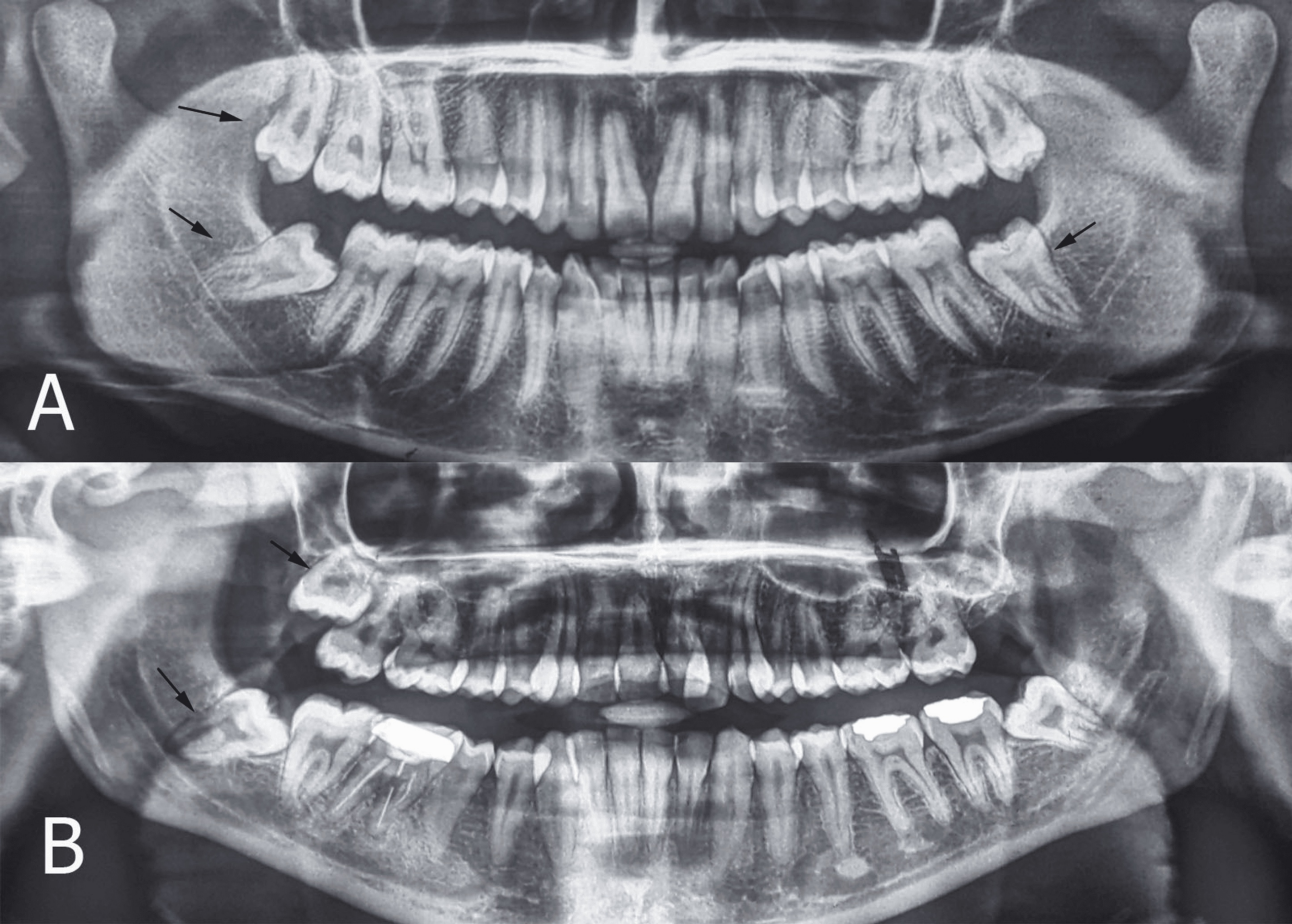
Orthopantomogram showing (A) impacted maxillary and mandibular third molar on the right side (difficulty index of 3 and 5, respectively) and (B) impacted maxillary and mandibular third molar on the right side (difficulty index of 5 and 6, respectively). Images are from patients included in the study, and the difficulty index was noted.

In a split-mouth study, 30 patients were divided into two groups: group 1 with maxillary impacted upper third molars requiring extraction, and group 2 with mandibular impacted lower third molars on the same side requiring extraction. Along with clinical examination, a preoperative orthopantomogram (OPG) was obtained for all the patients (Figure 1).

A thorough detailed history and various variables were noted pre-operatively, such as age, sex, closeness of the third molar to vital structures (maxillary sinus for upper third molars and mandibular canal for lower third molars), root curvature, crown width of third molars in comparison with adjacent second molars, mouth opening, and detailed medical history. To determine the surgical extraction difficulty pre-operatively, the criteria set forth by the Pederson index [11], where the type of impaction, depth of impaction, and space available, were scored to assess the surgical difficulty. It is an internationally recognized, standardized, and validated metric for assessing difficulty, with scores ranging from 7 to 10 signifying a high level of difficulty, scores from 5 to 7 representing a moderate level of difficulty, and scores between 3 and 4 indicating a low level of difficulty.

Patients were chosen at random, with the extraction of the lower third molar performed initially, followed by the extraction of the upper third molar; conversely, the extraction of the upper third molar was conducted first. All surgical extractions were performed by a single, skilled practitioner. Using local anesthesia, a triangular flap was created, and after sufficient bone removal, elevators were employed for tooth extraction, unless it was necessary to perform tooth segmentation. The total duration of the surgical procedure was recorded in minutes, from the initial incision until the final suture was placed. The technique required for the extraction of impacted molars was also noted (bone removal and use of elevators or bone removal with tooth segmentation). After the extraction of the third molar, a second impaction was planned after one week for the same patient.

All clinical assessments were performed by a trained and experienced evaluator, and all radiographic assessments were performed by three experienced and calibrated evaluators. To assess the intra-evaluator reliability of the radiographic assessments, 10 OPGs were re-evaluated at two-week intervals, and an intraclass correlation coefficient (ICC) of 92% showed high reliability and reproducibility. Inter-evaluator reliability (ICC value of 89%) also showed high reproducibility and reliability.

Statistical analysis

Statistical analysis was conducted using SPSS, Version 22.0 (IBM Corp., Armonk, NY). The Shapiro-Wilk test was used to assess data normality. Continuous variables are presented as mean ± standard deviation, while nominal data are reported as frequencies and percentages. Chi-square analysis was used to evaluate the associations between categorical variables, and an independent t-test was performed to determine the significance of differences between the groups. Descriptive and bivariate analyses were performed, followed by model calibration for each predictor variable. Initially, the models were calibrated for each predictor, incorporating all independent variables, with a significance threshold set at p ≤ 0.05.

## Results

The mean patient age was 29.93 ± 2.81 years. The mean mouth opening was 41.25 ± 3.5 mm. Chi-square test results revealed no significant differences between the maxillary and mandibular groups across all variables. Root curvature was absent in 22 (37%) cases and present in 8 (13%) cases in both groups (p = 1). Root fusion, vital structural proximity, and crown size also showed no significant differences, with p-values of 0.602, 0.273, and 0.602, respectively. The distance from vital structures such as the maxillary sinus in the upper arch was >5 mm in 22 (37%) cases, whereas in the mandibular arch, the distance from the mandibular canal was less than 5 mm in 12 (20%) cases and > 5 mm in 18 (30%) cases. In the maxillary arch, mesio-angular was the most common type of impaction, found in 12 (20%) cases. Similarly, spatial arrangement, depth, space available, and sex distributions were comparable between the groups (p > 0.05). Lastly, no significant difference was observed in the difficulty level between the groups, with slight, moderate, and very difficult cases distributed similarly (p = 0.301). In the maxilla, most impactions were slightly to moderately difficult, whereas in the mandibular arch, most impactions were moderately difficult (Table 1).

**Table 1:** Descriptive analysis and analysis of association between variables using the chi-square test. p > 0.05: non-significant.

Variables	Category	Maxillary third molar	Mandibular third molar	Chi-square test (p-value)
Root curvature	Absent	22 (37%)	22 (37%)	1
	Present	8 (13%)	8 (13%)	
Root fusion	Free	18 (30%)	16 (27%)	0.602
	Fused	12 (20%)	14 (23%)	
Distance from vital structures (mm)	<5	8 (13%)	12 (20%)	0.273
	>5	22 (37%)	18 (30%)	
Crown size	Bulbous	12 (20%)	14 (23%)	0.602
	Non-bulbous	18 (30%)	16 (27%)	
Type of surgical technique	Ostectomy + elevators	18 (30%)	16 (27%)	0.602
	Ostectomy + tooth sectioning	12 (20%)	14 (23%)	
Spatial arrangement	Mesio-angular	12 (20%)	8 (13%)	0.712
	Vertical	6 (10%)	6 (10%)	
	Horizontal	6 (10%)	8 (13%)	
	Disto-angular	6 (10%)	8 (13%)	
Depth	Level A	18 (30%)	16 (27%)	0.817
	Level B	6 (10%)	8 (13%)	
	Level C	6 (10%)	6 (10%)	
Space available	Class 1	20 (33%)	18 (30%)	0.412
	Class 2	6 (10%)	10 (17%)	
	Class 3	4 (7%)	2 (3%)	
Sex	Male	14 (23%)	16 (27%)	0.606
	Female	16 (27%)	14 (23%)	
Difficulty level	Slight difficult	12 (20%)	8 (13%)	0.301
	Moderately difficult	12 (20%)	18 (30%)	
	Very difficult	6 (10%)	4 (7%)	

The independent t-test results revealed significant differences between the maxillary and mandibular groups in procedure duration. The duration of the procedure was significantly longer in the mandibular group (44.13 ± 15.20 minutes) than in the maxillary group (33.33 ± 13.96 minutes), with a p-value of 0.006. No significant differences were found between the groups in terms of the difficulty index (p = 0.394), as shown in Table 2. Therefore, the null hypothesis was accepted in our study.

**Table 2:** Comparison of continuous variables between groups using the independent t-test. *p ≤0.05: significant. CI, confidence interval; SD, standard deviation

Variables	Group	N	Mean ± SD	95% CI of mean		p-Value
				Upper	Lower	
Duration of surgical procedure (minutes)	Group 1	30	33.33 ± 13.96	38.549	28.117	0.006*
	Group 2	30	44.13 ± 15.20	49.811	38.456	
Difficulty index	Group 1	30	5.26 ± 1.98	6.007	4.527	0.394
	Group 2	30	5.66 ± 1.60	6.266	5.068	

Multivariate regression analysis revealed multiple significant predictor variables for the difficulty index. Specifically, root curvature (p = 0.049), duration of the surgical procedure (p = 0.014), type of surgical technique employed (p = 0.042), and patient sex (p = 0.005) were identified as significant factors. In particular, both the root curvature and surgical technique adversely affected the difficulty index, whereas the duration of the procedure and sex exhibited positive coefficients, suggesting a direct correlation. This indicated that the difficulty index was elevated in females, and as the difficulty index increased, the duration of the surgical procedure also increased. Furthermore, an increase in the difficulty index necessitates ostectomy and tooth sectioning for removal. Similarly, the curvature of the roots contributed to a heightened difficulty index. Conversely, mouth opening, root fusion, and tooth impaction were not significantly correlated with the difficulty index (p > 0.05). Despite being non-significant, it was suggested that greater mouth opening, fused roots, and maxillary impactions were linked to reduced difficulty. Additionally, the constant of the model was significant (p = 0.01) with a 95% confidence interval ranging from 1.82 to 13.09 (Table 3).

**Table 3:** Multivariate regression analysis of predictor factors for difficulty index. *p ≤ 0.05: significant. As constant is not associated with any specific independent variable, the coefficient column for the constant is left blank because there is no specific predictor factor to display.

Model	Coefficients	Standard error	t-Value	p-Value	95% confidence interval	
					Lower bound	Upper bound
Constant	-	2.81	2.65	0.01*	1.82	13.09
Mouth opening (mm)	-0.16	0.06	-1.28	0.206	-0.21	0.05
Root curvature	-0.02	0.52	-0.12	0.049*	1.1	0.97
Roots fusion	0.23	0.5	1.65	0.105	-0.18	1.83
Duration of procedure (minutes)	0.28	0.02	1.48	0.014*	-0.01	-0.08
Type of surgical technique	-0.14	0.63	-0.8	0.042*	1.77	0.76
Sex	0.37	0.45	2.96	0.005*	0.43	2.22
Impacted tooth	-0.06	0.47	-0.45	0.651	-1.15	0.73

## Discussion

Due to the lack of studies comparing the difficulty index for impacted third molars between the maxillary and mandibular arches, this split-mouth study was conducted. The results of the present study indicated that there was no significant difference in the difficulty index between the arches. Owing to the lack of studies, this finding could not be directly compared. According to a study by de Carvalho et al. [10], the surgical difficulty of MTMS was low. The reason for the similar difficulty index observed in our study might be due to the non-significant difference in the variables that affect the surgical procedure, such as the distance of vital structures, root fusion and curvature, available retromolar space, depth of impaction, and crown size. All extractions were performed by the same experienced oral and maxillofacial surgeon.

The results of our study further revealed that the duration of the surgical procedure was longer in the mandibular arch than in MTMS. This could be because the mandible exhibits a greater density and compactness of the cortical bone than the maxilla [12]. In cases involving the mandible, impacted third molars frequently lie in proximity to or in direct contact with the inferior alveolar, as observed in our study. Consequently, heightened caution is imperative during the extraction process to mitigate the risk of nerve damage, which may extend the duration of the surgical procedure [6]. The mandibular arch is enveloped by a more robust and denser musculature (for instance, the masseter and pterygoid muscles), thereby complicating access and manipulation during surgical intervention. Conversely, the maxilla is characterized by a lower concentration of adjacent muscle mass, potentially facilitating surgical navigation. Similar results were reported in a systematic review by Akadiri and Obiechina [13].

Our study indicated that root curvature, duration of the surgical procedure, type of surgical technique employed, and patient sex were all significant predictors of surgical difficulty encountered during third molar extractions. Bede concluded that the duration of the procedure was a significant indicator of the difficulty of third molar extraction [14]. Females typically exhibit smaller mandibles and narrower dental arches than their male counterparts do [15]. This anatomical difference may result in insufficient space for the proper eruption of the third molars, consequently increasing the likelihood of severe or complex impactions. Limited spatial availability could exacerbate the challenges associated with extraction, as the teeth may be more densely situated or in proximity to critical anatomical structures. Furthermore, females generally demonstrate a lower bone density than males, particularly with advancing age. Although this could ostensibly imply that extraction procedures are less challenging, diminished bone density can complicate surgical intervention. This is attributable to the increased susceptibility of the bone to fractures or other complications during the extraction process [15].

Although the difficulty index showed non-significant differences between both arches, the multivariate analysis revealed that the maxillary arch was associated with lower difficulty compared to the mandibular arch. This finding is supported by those of previous studies [4,10]. Furthermore, the curved roots of impacted third molars are positively associated with surgical difficulty. When root structures exhibit pronounced bends or curvatures, they tend to deviate from a typical extraction trajectory with greater difficulty. This complexity necessitates the implementation of supplementary methodologies by the surgeon, which may include the segmentation of the dental structure into diminutive fragments.

Clinical implications

Although surgical difficulty for impacted third molars was found to be similar in both arches, extra care has to be taken while extracting the impacted third molars in mandibular arch, particularly the teeth with curved roots, proximity to the mandibular canal, and in females. In cases of curved roots close to the mandibular canal, it would be advisable to perform tooth segmentation and remove it in pieces.

Limitations

The small sample size may be one of the limitations of our study. We used the Pederson index to evaluate surgical difficulty, which has its own limitations; other indices were not considered in our study. Furthermore, the effect of mouth opening was not evaluated, as only patients who had normal mouth opening were selected. In future research, it is imperative to establish a rigorous correlation between diverse categories of difficulty indices and clinically validated methodologies for the evaluation of difficulty levels.

## Conclusions

Based on the findings of the present study, it was concluded that the difficulty index for impacted third molars was similar in both the arches. The third molars showed slight-to-moderate difficulty in the upper arch and moderate difficulty in the lower arch. Duration of the surgical procedure, root curvature, sex, and type of surgical technique were significant predictors of the difficulty index. Females with curved lower third molar roots were significantly associated with heightened difficulty in extraction, required ostectomy with tooth sectioning, and increased surgical duration.
